# 

**Published:** 1890-06-14

**Authors:** 


### NOTICE TO CORRESPONDENTS.

All MS., Letters, Books for Review, and other matters intended for the Editor should be addressed The Editor, The Lodge, Porchester Square, London, W.

The Editor cannot undertake to return rejected M.S., even when accompanied by stamped directed envelope.

**Notice to Advertisers and Subscribers.**

All Advertisements, Orders for Copies of The Hospital, and Business Communications should be addressed to The Publisher, *not to the Editor*, The Hospital, 140, Strand, W.C.

**Bound Volumes of "The Hospital."**

Vol. VI., containing full page portraits of T.R.H. the Prince and Princess of Wales, and Miss Florence Nightingale, is obtainable at 4s., post free.

Orders will be received for Vol. VII., 4s. post free. Cases for binding may be had of the Publisher, at 1s. 6d. each.

**To Residents, Secretaries, and Matrons.**

The Editor will be glad to have the Regulations and each Report as issued by Asylums, Hospitals, Convalescent and Nursing Institutions, as well as those of other Charities, and an early copy in duplicate of all Appeals and Festival Notices, etc., addressed to *The Editor, The Lodge, Porchester Square, London, W.* Any superintendent, secretary, matron, or other chief official who regularly sends such information may be registered, on application to the Publisher, to receive free of cost a cover for binding The Hospital in half-yearly volumes.

### BURDETT'S HOSPITAL ANNUAL FOR 1890

Is now ready, and may be obtained of the Publisher, The Hospital Offices, 140, Strand, W.C., price 2s. 6d. limp boards, or 3s. 6d. cloth. All orders must be accompanied by a remittance.

### Scraps and Gleanings.

Cholera is reported to be prevalent at Bagdad.

Hospital Saturday at Bath saw the collection of £294.

Harlington Cottage Hospital treated 29 patients last year.

An infectious hospital of three pavilions is to be erected at Walthamstow.

The Emperor has contributed 20,000 marks to the new German Hospital in Zanzibar.

Sleaford Working Men's Hospital Committee have got £135 by their annual collection.

Girton students have been attending some very successful ambulance classes held in Cambridge.

Dr. William Hickling has been specially commended for his coolness when the *Dacca* foundered.

Southampton Hospital Sunday Fund has reached £1,183; the largest collected in that town.

A Magnificent bazaar in aid of the Children's Home at Luton has been held and £533 was taken.

The Duchess of Albany distributed the certificates to the Oxford corps of the St. John's Ambulance Association.

At Grosvenor House, on June 5th, a fashionable concert was given in aid of the London Homœopathic Hospital.

The London Skin Hospital issues a special appeal for funds, as increasing work necessitates moving into larger premises.

The Prince of Wales will lay the foundation stone of the Royal South London Ophthalmic Hospital, new buildings in July,

Dumfries infirmary report still tells of a too great expenditure, though an enquiry has lately been held on the subject.

A ladies' Committee seems needed at Macclesfield where the separate duties of the matron and house surgeon are not properly defined.

The Metropolitan Convalescent Institution, while benefitting by experience of half-a century, has kept pace with the requirements of the day.

Heywood Brothers, of Manchester, have patented an automatic disinfecting closet, which ought to secure the attention of hospital authorities.

Mr. H. Francis has been appointed Assistant House Surgeon at the Female Lock Hospital, Harrow Road, in place of Mr. H. C. L. Morris, resigned.

Mrs. Handley laid the foundation stone of the new children's wing of the Royal United Hospital, Bath, lately. The new wards will hold twenty beds.

Some excellent sermons were preached in London on Hospital Sunday, notably those of the Ven. W. M. Sinclair, at St. Paul's, and Dr. Vaughan at the Temple.

The Convalescent Home for Children, started at Bushey Heath by Miss Derham and Miss Dunn, has removed into larger premises at Caldicote House.

The memorial stones of the new children's ward to Burnley Hospital, were laid by Mr. Altham, Mr. E. Ecroyd, and Lady O'Hagan, in the presence of 2,000 spectators.

The Prince of Wales has accepted the post of honorary president of the International Congress of Hygiene and Demography which will be held in London next year.

"A Little Friction" is an often prescribed remedy in hospitals, but it is to be regretted that it was present at Brighton at the meeting of the Hospital Saturday Committee.

At the approaching Encænia of the University of Oxford, the honorary degree of D.C.L. will be conferred on Sir William Turner, Professor of Anatomy at Edinburgh University.

A medical student of Guy's Hospital, was charged, at Southampton, with being a stowaway. He had been ordered a sea voyage, and went to the Cape, but had no money to get home with.

Cork North Infirmary treated 916 patients last year, and unfortunately contracted debt in the course of its duties. We wish it a rapid recovery to its normal healthy state of finance.

The promoters of the Infectious Diseases Bill have decided to abandon Clause 6 of that measure, requiring laundrymen to give up a list of their customers on the demand of the medical officer of health.

The building fund for Salisbury Infirmary is progressing splendidly. Sir Edward Hulse has given £100, and the Earl of Pembroke £500. Lord Radnor is working hard in the interests of the fund.

For Scotch caution carried to a most selfish excess, commend us to the dwellers at Aboyne! A lady has offered to build a convalescent home for children there, but the inhabitants have petitioned the Marquis of Huntley not to grant a site for such an object. For shame, Aboyne!

Western Infirmary, Glasgow.—By the munificence of Colonel Hozier, of Mauldslie Castle, a convalescent home, with land attached, situated in the Lanark district, has been given to the infirmary. The building in question was originally fitted, at a cost of £5,000, for use as a military store-house.

The Alexandra Hospital (so called by permission of the Princess of Wales), at Woodhall Spa, was formally opened on the 29th ult. by the Countess of Brownlow. The ceremony was attended by a large and distinguished company from all parts of the county. There is accomodation for from twenty to thirty poor patients.

After Care Association for Poor and Friendless Female Convalescents on Leaving Asylums for the Insane.—The annual meeting will take place at 83, Lancaster Gate, W., on June 23rd, at 3 p.m. The Earl of Meath will preside. Cards of invitation can be obtained of the Secretary, Mr. H. Thornhill Roxby, Arden Lea, The Drive, Walthamstow, Essex.

The Carnival bazaar was very pretty and very crowded. The articles for sale were most moderate in price and varied in character. Miss Learmouth and her nurses took an active part at the sale and the medical staff work took an active part in the buying. We congratulate the North West London Hospital, which we are sure will benefit largely by this successful entertainment.

